# The challenges in diagnosis and management of osteitis pubis: An algorithm based on current evidence

**DOI:** 10.1002/bco2.127

**Published:** 2022-03-11

**Authors:** Mohammed Lotfi Amer, Kawa Omar, Sachin Malde, Rajesh Nair, Ramesh Thurairaja, Muhammad Shamim Khan

**Affiliations:** ^1^ Faculty of Medicine Tanta University Tanta Egypt; ^2^ Department of Urology Guy's and St. Thomas' NHS Foundation Trust London UK; ^3^ MRC Centre for Transplantation, Faculty for Life Sciences and Medicine, NIHR Biomedical Research Centre King's College London London UK

**Keywords:** chronic pelvic pain, osteitis pubis, osteomyelitis pubis

## Abstract

**Objective:**

The objective of this study is to summarise the contemporary evidence regarding the prevalence, diagnosis, and management of osteitis pubis (OP) specially from urological point of view, while proposing an algorithm for the best management based on the current evidence.

**Methods:**

We performed a literature search using the PubMed database for the term ‘osteitis pubis’ until December 2020. We assessed pre‐clinical and clinical studies regarding the aetiology, pathophysiology, and management of OP. Case reports and case series were evaluated by study quality and patient outcomes to determine a potential clinical management algorithm.

**Results:**

Osteitis pubis is a chronic painful condition of the symphysis pubis joint and its surrounding structures. Still, there is a paucity of data outlining the management plan and the possible triggers. The aetiology seems to be multifactorial with different proposals trying to explain the pathophysiology and correlate the findings to the outcome. The diagnosis is usually based on high suspicion index and clinical experience. The infective variant of the disease is aggressive and requires strict and active management. Universal consensus is still lacking regarding a formal algorithm of management of the condition, especially due to multiple specialities involved in the decision‐making process. Conservative management remains the cornerstone; nevertheless, surgical interventions may be needed in special settings. Hence, a multi‐disciplinary approach is of pivotal value in fashioning the plan for each case. The prognosis is usually satisfactory; however, a longstanding debilitating disease form is not uncommon.

**Conclusion:**

OP remains a rare condition with real challenges in its diagnosis. The current management is focused on conservative management; however, surgical intervention is still needed in some difficult scenarios. Continued research into the triggers of OP, multidisciplinary approach, and standardised clinical pathways can improve the quality of care for patients suffering from this condition.

## 1 INTRODUCTION

Osteitis pubis is typically a non‐infectious inflammatory painful chronic condition affecting the symphysis pubis and the surrounding structures. It is characterised by disabling pelvic and/or groin pain and a localised tenderness over the joint.^1^, ^2^

The very first report of this condition appeared in French literature in 1923 which was published by Legueue.^3^ Beer^4^ then published a small series of five cases in the English journal a year later in 1924 who developed the disease after different suprapubic procedures.

Aetiology of osteitis pubis is multifactorial. It may be caused by surgical procedures on any of the pelvic organs, surgery in the groin, treatment of urological conditions involving different energy sources, or radiation therapy. Osteitis pubis is also prevalent in athletes and patients with rheumatic diseases and pregnancy.

Irrespective of the causative factors, it is a difficult condition to treat. Although causes are diverse, in this review, we will principally focus on various urological procedures which can lead to this complication, diagnostic tests, and treatment strategies to manage the condition.

## 2 AETIOLOGICAL FACTORS

### 2.1 Urological

Osteitis pubis has been reported after a broad spectrum of urological procedures including transurethral resection of the prostate either monopolar or bipolar,^5^, ^6^ prostate cryotherapy, laser photo vaporisation of the prostate,^7^ periurethral collagen injection,^8^ transrectal needle biopsy of the prostate,^9^ high‐intensity focused ultrasound treatment of the prostate,^10^ radiotherapy, radical prostatectomy,^11^ and cystectomy.^12^

The conventional procedures for surgical treatment of stress urinary incontinence, specifically Marshall‐Marchetti‐Krantz (MMK) urethropexy, are the most cited procedure. MMK mandates inserting sutures primarily in the pubic rami's periosteum. Reviews of the MMK procedure cited osteitis pubis incidence in up to 2.5% of the cases, varying from 0.7% to 2.5%.^13^, ^14^, ^15^, ^16^

The case reports linking transurethral endoscopic prostatic surgery to osteitis pubis did not clearly identify the sequential evolution of the disease in these cases; however, some postulations were made. Excessive resection or lasing with capsular perforation may lead to sinus formation and infected urinomas, which ultimately lead to osteitis pubis.

Ziesel et al.^6^ identified 12 cases of osteitis pubis on a retrospective analysis of a cohort of 12 118 patients who underwent monopolar TURPs in a German institution. They analysed the possible factors that can provoke the disease, and they postulated that using a suprapubic trocar for irrigation during surgery might have induced the disease (11/12 cases). MRI imaging in 7 of the 12 patients demonstrated a sinus connected to the prostatic capsule. Authors identified only two visible perforations intraoperatively; however, they were not significant to warrant termination of the procedures. Hence, they proposed that most likely the energy applied to the prostatic tissue was the key factor in causation of the condition. Tissue necrosis due to impaired blood flow can result from hyperthermia or hypothermia or radiation injury to the prostatic tissue and its surroundings.^6^

This theory can explain the disease progression in many settings specially with the treatments of prostate cancer. For example, cryotherapy (It involves freezing), high intensity focal ultrasound, brachytherapy, and external beam radiation therapy all entail delivery of high energy to the prostatic tissue either thermal or radiational to eradicate cancerous tissues. Unfortunately, despite all trials to keep the effect limited to the targeted tissue, complications like rectal injury and urethrorectal fistula are still encountered in the practice.^6^ This can give a reflection towards what is really happening in osteitis pubis disease.

In a case report of a 68‐year‐old male without previous significant comorbidities who underwent Green Laser Photo Selective Vaporisation of the Prostate with a 120‐W Greenlight HPS for treatment of lower urinary symptoms due to an obstructive prostate, the authors reported that the prostate size was only 38 mls. This small size can give a clue to the progression of the disease later on, as the patient developed a calcitrant osteitis pubis which needed exploration eventually.^7^ The presence of a capsular perforation was confirmed at time of exploration; however, the authors did not have any data about the machine settings used during the lasing session. Despite that this laser has a high safety profile, nevertheless, the amount of energy delivered is high and powerful for the ablation purposes so that it can cause injury if not wisely used. This laser can form an area of a relatively low blood supply behind the target area of nearly 1 cm in length.^17^ This can lead to late necrosis of the tissues and sinus formation which can proceed to a subsequent well‐formed fistula. There are some reports in the literature for a prostate‐symphyseal fistula after endoscopic or laser prostatectomy.^18^, ^19^

The disease evolution after open radical surgeries, as example radical cystectomy or prostatectomy, can be explained by iatrogenic trauma either by instrumentations, longer duration of retraction, or by electrocautery which may cause tissue necrosis to the periosteum.^11^, ^12^

### 2.2 General surgery

In general surgery publications, abdominoperineal resection and inguinal herniorrhaphy were reported as possible triggers for the disease.^20^, ^21^

### 2.3 Sports medicine

Furthermore, this condition has been observed in athletic population, often with insignificant trauma, including long distance runners and ice hockey professionals. It also affects those involved in sports that require kicking, spinning, twisting, cutting, pivoting, sprinting, fast acceleration and deceleration, or abrupt changes in direction.^22^ The incidence of the condition in athletes has been recorded as 0.5–8%, with an increased incidence in marathon runners and in kicking sports, especially male footballers, accounting for 10–18% of reports every year.^2^

### 2.4 Rheumatological diseases

Some rheumatic diseases have been linked either directly or indirectly to osteitis pubis, particularly ankylosing spondylitis and rheumatoid arthritis. Resnick et al.^23^ have described osteitis pubis in 61% in a series of 18 women with ankylosing spondylitis. The existence of the HLA‐B27 antigen was used as an indicator for correlation with ankylosing spondylitis.

### 2.5 Gynaecology

The gynaecology literature also has some publications reporting osteitis pubis after a wide set of procedures. One of the early reports was documented by Painter^24^ following a traumatic vaginal delivery. Wiltse and Frantz^25^ reported the first series, which included 10 pregnancy‐related cases and 1 case after an anterior colporrhaphy. Moreover, although uncommon, osteitis pubis remains an obstetric problem both during the antepartum and postpartum periods.^26^

## 3 PATHOPHYSIOLOGY

Different aetiological and pathological factors contribute to the evolution of the osteitis pubis such as trauma, infection, and stagnation of venous blood in the surrounding venous plexuses. Although these factors can explain presentations of the condition in some individuals, they fail to interpret the evolution of the disease in others. Until now, the aetiology is still unclear and probably multifactorial.

### 3.1 Trauma

Trauma as the main trigger of osteitis pubis was hypothesised by Beer's^4^ pioneering work in 1920s. It sounds logical that injury to the symphysis pubis may lead to an inflammatory reaction that may involve the joint.

Trauma may be iatrogenic as in open pelvic operations caused by retraction, dissection, and thermal injury during haemostasis. As an example, in Marshall Marchetti Krantz (MMK) urethropexy entails direct insertion of sutures in the periosteum considered as local insult to the bone itself.

The other possible cause is exercise‐induced trauma. Osteitis pubis manifests in athletes as unexplained groin pain, due possibly to the repeated minor trauma. The estimated incidence ranges between 0.5% and 8%, with a relatively increased incidence in runners, kicking sports, football players, who represent approximately 10% to 18% of cases per year.^27^, ^28^ Microtears, pelvic girdle injuries specially loss of synchronisation between the abdominal adductor muscles and the hip structural mechanisms are currently considered the most fundamental pathogenic element in the evolution of osteitis pubis in this setting.^29^ This is expected in some sports which exhibit higher figures of the condition based on the rationale of rapid acceleration or deceleration injuries, specially running, kicking, and abrupt change of direction. Expectedly, soccer, fencing, American football, ice hockey, rugby, and cricket are commonly quoted sports.^2^, ^30^

In the contrast side, some actual trials were made to induce osteitis pubis by producing definitive iatrogenic trauma to the joint by different methods. Wheeler^31^ tried intended pubic trauma and infection in rabbits, however with unsatisfactory results. Another unsuccessful attempt by Beneventi and Spellman^32^ was made to provoke osteitis pubis in dogs through infection and other various insults, including the opening of the bladder to permit urine to enter the retropubic space of Retzius, and injury to the obturator nerve, fulguration of the joint periosteum, and cartilage excision. Although trauma is a very common occurrence, Osteitis pubis is rather rare, and hence, various factors may play an essential role in causation of this condition.

### 3.2 Infection

Low‐grade infection by less virulent organisms is another possible proposed mechanism causing osteitis pubis especially in postsurgical patients. In one series of osteitis pubis after MMK, seven patients failed conservative management and required surgery. Bone cultures from the surgical procedure were sampled and demonstrated infection in five patients (71%).^14^

In a series of four patients who underwent resection of the symphysis pubis, the finding of necrotic bony tissues was obvious. In addition, cultures of the urine and bone were bacteriologically similar. *Pseudomonas aeruginosa*, *E. coli*, and Bacillus proteus have been frequently isolated. The authors shed light on the correlation between the presence of an active urinary tract infection and the development of the symptoms with implications on the treatment plan.^33^

Coventry and Mitchell^34^ reported a series of 45 patients with osteitis pubis. In this cohort, bone material was collected for culture, but they found no organisms in this although pyuria with positive cultures was present in 16 of the 45 patients.

Limited histologic evaluation of osteitis pubis has been performed; nevertheless, few studies support inflammation as the core aetiological factor. In the series reported from the Mayo Clinic, seven patients had meticulous histopathological analysis. All showed an inflammatory exudate composed of plasma cells and lymphocytes, with evidence of marrow fibrosis and new bone formation in several samples.^34^

### 3.3 Neural irritation

Wheeler contended that osteitis pubis was a result of the continuous irritation of the pelvic sensory neurons secondary to an active infectious process resulting in permanent changes in the bony structure ‘causalgia‐like effect’ imitating what happens in Sudeck's atrophy or reflex sympathetic dystrophy disease.^31^ Nevertheless, Wiltse and Frantz^25^ proposed the localised reflex sympathetic dystrophy as the crucial step in the progression of the condition other than infection itself.

### 3.4 Thrombosis and venous stagnation

The hypothesis of venous thrombosis and congestion is based on the fact that the venous plexus drains some of the posterior veins of the pubic symphysis, and hence, obstruction of the venous system could cause hyperaemia with resultant bone demineralisation and necrosis. Due to the close association of the veins of the urinary tract and those that drain the pubic symphysis, and an anatomic lack of valves in these vessels, infection‐induced urinary stasis has also been proposed as an inciting factor for venous congestion.^34^ Steinbach et al.^35^ using osseous phlebography demonstrated obstruction to venous flow from the pubis in some of the patients who developed osteitis pubis, while absence of obstruction in those with no evidence of the condition.^35^, ^36^, ^37^

### 3.5 Avascular necrosis of the inter‐pubic disc

Avascular necrosis of the inter‐pubic disc can result either from traumatic damage to the blood supply or thrombophlebitis. Bone changes in the pubis are believed to result from avascular osteoporotic changes.

Turner Warwick proposed that postoperative osteitis pubis results from infective inter‐pubic disc necrosis, as fibrocartilage is fairly avascular and cannot survive infection; therefore, it initiates necrosis, and inter‐pubic abscess formation actually contains a soft‐tissue sequestrum, which should be excised to promote the settlement of osteitis pubis.^38^

## 4 CLINICAL FEATURES, PRESENTATION, AND DIAGNOSTIC WORK‐UP

There is a spectrum of presentations of osteitis pubis. Unexplained pelvic or groin pain is one of the key symptoms. History should focus on recent or previous urologic or pelvic surgical procedures, local trauma, or repetitive injury to the area in question and aggressive regular physical exercise as in athletics athletes.

Pain is usually localised in the lower abdomen, pubis, or groins. It is generally dull, aching, or throbbing in character and eases with rest. Pain is either acute or subacute in onset (approximately 6 to 8 weeks after an offending surgical procedure) with chronic progressive course (3–12 months) and finally remission in most of cases.

Generally, it is mild to moderate discomfort but can become severe throbbing pain during the physical activity. It may radiate to the inner thigh and adductor muscles on the affected side(s). Pain is aggravated on physical activity that increases pressure on the pelvic girdle such as walking, coughing, sneezing, lying on the side, and either climbing up or walking down the stairs. Mitigating factors include rest, pain killers, and anti‐inflammatory drugs. It may result in gait disturbances and result in waddling gait when patients walk with legs spread apart. Constitutional symptoms are uncommon, but some patients may occasionally experience low grade fever and malaise.

Physical examination should focus on excluding the groin hernias and prostate examination in men to rule out prostatitis; in women, a thorough pelvic examination to rule out other diagnoses such as pelvic inflammatory disease should be performed. Local physical examination may reveal localised tenderness over the pubic symphysis or lateral to the pubic symphysis. More specific tests to make a clinical diagnosis include ‘public spring’ test and ‘lateral compression’ test, whereas helpful tests that may reproduce symptoms include the FABER (flexion, abduction, and external rotation) test and the ‘adductor squeeze’ test.^1^, ^26^, ^39^

## 5 LABORATORY TESTS

In few cases, inflammatory markers like erythrocyte sedimentation rate ESR and C‐reactive protein CRP may be slightly raised, though these are nonspecific markers. In patients with fever and feeling generally unwell, blood cultures and a complete blood count should be checked. If blood culture prove negative, aspiration and culture of the joint space may be beneficial to isolate any organisms.^40^ Because of the possible association with urinary tract infection, a clean‐catch urine culture can also be sent to the lab.^33^

## 6 IMAGING

Different radiologic modalities can be used for diagnosis or exclusion of the osteitis pubis (Table 1). Imaging modalities include plain radiographs, magnetic resonance imaging (MRI), bone scintigraphy, and symphysography.^1^, ^26^, ^39^

**TABLE 1:** The diagnostic characteristics of osteitis pubis in different imaging modalities^1^

Imaging modality	Findings
X‐ray of the symphysis pubis^25^, ^31^, ^33^, ^34^, ^37^, ^38^	Joint irregularities Sclerosis Osteophytes formation on the articular surfaces Widening of the pubic symphysis joint space
MRI^41^	**Acute** (less than 6 months) • Periarticular oedema • Fluid in the pubic symphyseal joint • Bone marrow oedema **Chronic** (more than 6 months) • Subchondral sclerosis • Resorption • Osteophytes • suprapubic fistulae
Scintigraphy scans^42^	Focal accumulation of nucleotide tracer at or around the pubic symphysis • Unilateral or bilateral uptake
Cleft injection phlebography (Symphysography)^43^	Loss of disc morphology Extravasation into local bony defects Lymphatic/venous intravasation from hyperaemia (less common)

### 6.1 Conventional X‐ray radiographs

Plain radiographs may appear normal in the early stage.^1^ In the well chronic established disease, the pubic symphysis may show either lytic changes or sclerosis and widening of the pubic symphysis.^25^, ^31^, ^33^, ^34^, ^37^, ^38^ Dynamic instability of the pubic symphysis can be seen on a special view called “flamingo view” which is a specialised orthopaedic series consisting of three separate pelvis projections (neutral, right foot raised, and left foot raised). It is used for assessing instability of the pubic symphysis, often in the context of previous pelvic trauma.^2^, ^44^

### 6.2 Magnetic Resonance Imaging

Magnetic Resonance Imaging (MRI) is the imaging of choice when osteitis pubis is suspected.^41^, ^45^ MRI has a high sensitivity in differentiating between acute and chronic OP.^1^, ^26^, ^30^, ^39^ In the acute setting, MRI demonstrates subchondral oedema of the bone, typically affecting both sides. The changes in chronic OP include periosteal reaction, bone resorption, irregularity of the articular surface, osteophytes, and subchondral cyst formation. Bone marrow oedema of the pubic symphysis is also commonly reported in asymptomatic cases. A linear subchondral T2 signal, parallel to the pubis, is commonly found in patients who are heavily symptomatic.^46^

Changes on MRI usually correlate with the clinical outcomes of osteitis pubis. Presence of oedema in the pubic bone as well as surrounding musculature is a poor prognostic sign with a lower probability of complete recovery. On the contrary, if oedema only involves the bone, then there are better prospects of recovery at 18 months.^47^ Finally, MRI is also useful for diagnosing concomitant injuries that can contribute to osteitis pubis, such as adductor tears, femoro‐acetabular impingement, rectus femoris tears, and labral tears.^39^, ^41^

### 6.3 Bone scintigraphy

Bone scintigraphy is a highly sensitive imaging tool using radionuclide tracers. It helps in detection of bony lesions by recording abnormal osteoblastic activity due to various whether caused by infection, trauma, or metastatic cancers.^48^ Kalawat et al.^42^ reported findings of bone scintigraphy in a 58‐year‐old female who complained of lower abdominal and pain in the left side of the hip joint. The findings confirmed traumatic osteitis pubis and early arthritic changes in the left Sacro‐iliac joint, which were subsequently confirmed by CT fusion technology.

### 6.4 Symphyseography

O'Connell et al.^43^ evaluated the role of intra‐symphyseal cleft injection for diagnosis and treatment of 16 professional athletes with clinically suspected osteitis pubis referred over a 6‐month period. Symphysography was performed under fluoroscopic guidance under strict aseptic conditions (Table 2). Under local anaesthesia, an access needle was advanced into the symphyseal cleft at a point midway between the upper and lower borders of the symphysis. The needle was then further advanced nearly 1 cm into the cleft of the fibrocartilaginous disk. After positioning of the needle, 1 ml of nonionic contrast material was injected into the symphyseal cleft to confirm appropriate position, show the structure of the disk, and potentially elicit the pain. A plain anteroposterior X‐ray film recorded the morphology of the disk. Subsequently, injection of a solution composed of 20 mg of methylprednisolone acetate and 1 ml of 0.5% bupivacaine hydrochloride was processed into the cleft aiming for reducing the inflammatory reaction and relieve refractory pain.

**TABLE 2:** The following table illustrates the findings of symphysography in the work done by O'Connell et al.^43^

Finding	No of patients	Percentage
Symmetrical marginal sclerosis of the medial margins of the pubic bones	14	87.5%
Marginal erosions	9	56.2%
Marginal osteophyte formation	5	31.2%
Widening of the joint space and poorly defined cortical margins	2	12.5%
Joint disruption with malalignment of the pubic bones	2	12.5%

## 7 DIFFERENTIAL DIAGNOSIS

The differential diagnosis comprises a wide variety of conditions (Table 3).

**TABLE 3:** The list of conditions to be considered in the differential diagnosis of osteitis pubis^2^

Other causes of intra‐abdominal pain	Other causes of musculoskeletal pain
Prostatitis	Osteomyelitis of the symphysis pubis
Urinary retention	Adductor tears
Ureteric colic	Adductors tendinopathy
Obstructed or strangulated inguinal or femoral hernias	Posttraumatic benign pubic osteolysis
Appendicitis	Malignant metastases
Colonic diverticulosis	Femoro‐acetabular impingement syndrome
Adenexial masses or torsions	Acetabular labular tears
Ovarian cystitis or ruptured ovarian cyst	Transient synovitis
Salpingo‐oophritis or ectopic pregnancy	Pubic ramus stress fractures
Pelvic inflammatory disease or endometriosis	Greater trochanter pain syndrome
Irritable or inflammatory bowel diseases	Groin pain disruption
Inguinal lymphadenopathy	Nerve entrapment syndromes

It is important to differentiate between osteitis pubis and osteomyelitis of the pubic bone (Table 4). The differentiation between these two may be challenging due to similarities in presentation. The most common complaint in both is suprapubic/groin pain specially with any stress on the pelvic girdle musculature as lifting heavy objects. The laboratory investigations are usually normal or marginally abnormal in osteitis pubis but more significant increase in inflammatory markers in osteomyelitis. Bone scan may show increased activity in the mineralisation or delayed phase in case of osteitis pubis; however, the profound activity is obvious in all the three phases in case of osteomyelitis. Aspiration and culture of the aspirate are the diagnostic test which establishes the diagnosis even after antibiotic therapy has been started.^49^, ^50^

**TABLE 4:** Differences between osteitis pubis and osteomyelitis of symphysis pubis^49^

	Osteomyelitis of the symphysis pubis	Osteitis pubis
Other synonyms	Infectious osteitis pubis Osteitis pubis purulenta	Non‐infectious osteitis pubis Inflammatory osteitis pubis
Past history	Recent pelvic surgery (Urologic, gynaecologic, or general surgery) Spontaneous trauma (athletics) TRUS guided prostate biopsy IV Drug users Ankylosing spondylitis and rheumatic diseases	
Presentation	Suprapubic or groin pain Limitation of movements with classic waddling gait	
Constitutional symptoms	High grade fever Toxic look and generalised malaise and fatiguability	Low grade fever (occasional) Generally well patients
Examination	Tenderness over suprapubic area Special tests: Spring test and FABER test can reproduce the symptoms	
Laboratory findings	High inflammatory markers (CRP, ESR) Increased WBCs count	Low or normal inflammatory markers
Radiology	May detect retropubic abscesses or collections in the surrounding structures	Degenerative changes in chronic cases
Joint aspiration and culture	Positive with bacterial growth Mainly high virulence bacteria as Staph aureus, *pseudomonas aeruginosa* and Klebsiella.	Usually negative Sometimes low virulent organisms
Histology	Active bacterial infection process	Purely inflammatory process with plasma cell and lymphocyte infiltrates
Conservative treatment	Antibiotics Analgesics	Rest NSAIDS Corticosteroids Anticoagulants
Surgical treatment	Surgical intervention is usually needed to drain abscesses or collections	Limited to cases with failed conservative measures
Prognosis	Serious condition and needs active management	Good recovery in more than 90% of cases

## 8 TREATMENT

Much of our knowledge about the treatment of osteitis pubis comes from the sports medicine literature because higher rates of osteitis pubis are seen in the athletes. Treatment modalities range from conservative management with rest to invasive surgical interventions. Although urologists can introduce conservative treatment, but if these measures fail, then referral to an orthopaedic/sports medicine specialist is most appropriate. The role of physiotherapy may be beneficial specially in athletes. Due to the rarity of this condition, no well‐conducted studies have been carried out to determine the best treatment pathway.^1^, ^51^

### 8.1 Conservative management

#### 8.1.1 Supportive measures

Conservative methods include a short period of bed rest followed by gradual and progressive ambulation with or without the use of assistive devices such as crutches or a cane. Minimising activities that place stress on the pelvic girdle should also be recommended during this phase of treatment. Local application of hot or cold therapy over the pubic symphysis can also be employed. The use of specially designed compression shorts with the aim of limitation of the groin pain has also been tried with modest results.^52^

#### 8.1.2 NSAIDS

Oral nonsteroidal anti‐inflammatory agents such as ibuprofen or cyclooxygenase‐2 inhibitors can be used.^1^, ^51^ Addition of physiotherapy and rehabilitation programs at this stage may improve the symptoms and decrease the time till return to the normal activity.^1^, ^51^ Use of Phenylbutazone, a nonsteroidal anti‐inflammatory drug (NSAID), resulted in good response in five of six patients with osteitis pubis.^20^ When inflammation is severe, the use of opioid based analgesics may be required for adequate symptomatic relief.

#### 8.1.3 Corticosteroids

Glucocorticoids have played a pivotal role in the management of array of inflammatory diseases including and osteitis pubis. Steroid therapy has been used both orally and through parenteral route. Treatment with adrenocorticotrophic hormone (ACTH) was first reported by Marshall et al.^53^ for osteitis pubis that developed after urologic procedures. Thereafter, use of cortisone preparations has been reported in several publications with good results.^36^, ^54^, ^55^ However, steroids with minimal mineralocorticoid effects such as dexamethasone are usually the preferred choice. A maximum daily dose of 8 to 10 mg of dexamethasone provides a dramatic response in the symptoms, particularly pain, within 24 to 48 h. The duration of therapy has not been specified but has to be tailored according to severity of symptoms with a 10‐ to 14‐day course usually sufficient in mild cases while longer protocols of treatment may be needed in severe cases.^26^

#### 8.1.4 Local injection therapy

When conservative measures fail, corticosteroid injection (dexamethasone, betamethasone, and methylprednisolone) with or without adjuvant anaesthetic (bupivacaine and lidocaine) into the pubic symphysis can be tried. This has been shown to be effective with rapid reduction in most patients.^1^ There is no consistency in the reports of the type or dose of the corticosteroid preparations administered. A well‐conducted meta‐analysis showed that nearly 60% of athletes treated with corticosteroid injections were able to resume their activities; however, there is still approximately 20% without any documented improvement.^51^ As previously mentioned, symphysiography or symphyseal cleft injections can be both valuable from both diagnostic and therapeutic perspectives if corticosteroid injection is utilised at the time of this diagnostic procedure.^43^

#### 8.1.5 Bisphosphonates

The use of IV pamidronate/Bisphosphonates which are synthetic pyrophosphate analogues that have shown to be effective in a variety of metabolic disorders of bone was reported in a case series to be a safe and an efficient treatment option for patients with resistant osteitis pubis.^56^

#### 8.1.6 Anticoagulants

There are very limited data regarding the use of anticoagulants in the treatment of osteitis pubis. The basis of this treatment is the theory of venous congestion or thrombosis as the underlying cause of OP in some patients. The role of heparin was first suggested by Mynors,^57^ but this was refuted by Nissenkorn et al.,^58^ who claimed that there is no role of prophylactic administration of heparin in preventing the development of osteitis pubis. This was based on the clinical use of heparin in seven patients and the observation of no obvious improvement except in only two.^58^

In one early small series of three postoperative patients (one following prostatectomy and two following vaginal delivery), treatment success was only seen after heparin therapy.^37^ Later, another small series of three patients with osteitis pubis following prostatectomy, conservative measures failed, but clinical improvement was only seen with initiation of intravenous heparin therapy.^59^

There is only a single case report of successful resolution of symptoms with warfarin therapy for 6 months in a patient with intractable pubic symphyseal pain following uncomplicated retropubic prostatectomy for benign prostate hyperplasia.^60^

#### 8.1.7 Other treatment options

The use of antibiotics has been advised by proponents of the infection theory. External beam radiation has also been utilised for this condition.^61^ Coventry and Mitchell^34^ documented remission of symptoms in nine patients using radiation therapy. However, in the current practice, radiation is not considered as a treatment option because of the potential long term carcinogenic effects. Anecdotal use of vitamin B and diathermy has also been reported.^62^

### 8.2 Surgical management

Evidence suggests that over 90% of patients respond to medical management and surgical treatments are only given a consideration in a minority of patients in whom conservative therapy has failed patients have disabling symptoms (5–10%).^2^, ^63^ Generally, surgical interventions are only considered after a minimum of 6 months of conservative treatment.^64^ Because of the limited number of cases requiring surgical intervention, there is no consensus on the best surgical approach.^1^

Surgical options include the following:

- curettage of symphyseal fibrocartilage and removal of necrotic bone;
- arthrodesis and wedge or wide resection of the pubic symphysis with a wide range of procedures to strengthen or fix the abdominal or pelvic floor muscles.^39^

Samellas and Finkelstein^33^ reported open exposure and curettage for four patients with osteitis pubis. Following the procedure, there was swift resolution of symptoms. Cibert^65^ also reported good outcomes in 11 of 15 patients with excision of the granulation tissue and drainage. Warwick^38^ described two cases of osteitis pubis treated by synchondrectomy (exposure and curettage) with excellent outcomes.

Wedge resection of the joint is another surgical option for selected nonresponding cases. Grace et al.^63^ reported the use of this technique in 10 patients with osteitis pubis who did not show any improvement on conservative treatment. All patients had significant improvement after an average of nearly 12 months. However, during subsequent follow up, 3 of the 10 patients had relapse of symptoms. For such patients, they recommended symphyseodesis and fixation with a compression plate.^63^

These techniques are clearly invasive and not without complications. Wide resection of the pubic symphysis can cause complications like pelvic instability requiring additional surgical procedures.^66^ Mehin et al.^67^ after reviewing a case series of 10 patients and review of the literature recommended curettage of the joint for less complex cases and wedge resection in those suspected to have residual infection on curettage after urologic surgery. More recently, endoscopic techniques have been successfully demonstrated in case reports.^68^, ^69^

### 8.3 New Novel approaches

Few other treatments strategies have been proposed. These include pulse‐dose radio frequency,^70^ distal rectus abdominis ultrasound‐guided needle tenotomy and platelet‐rich plasma injection,^71^ and extracorporeal shock wave therapy (ESWT).^72^ Another question about the role of hyperbaric oxygen therapy (HBOT) has been studied in management of chronic osteomyelitis with some promising results,^73^, ^74^ in management on challenging cases of osteitis pubis. The idea is to put the patient in a room with a high oxygen concentration (100%) under high atmospheric pressure for nearly 90 min a day for average of 1–2 months. This may act by potentiating leukocyte oxidative killing, osteogenesis, angiogenesis, and synergistic antibiotic activity.^75^ As the data on these newer modalities are rather limited, further studies are needed to assess the efficacy of these newly introduced therapies.

### 8.4 Prognosis

The prognosis of osteitis pubis is reasonable, as 90% to 95% cases resolve with conservative therapies.^76^, ^77^ Nevertheless, the duration of recovery period is unpredictable. Fricker et al.^78^ documented an average of 9.5 months to full recovery in men (with a range from 3 weeks to 48 months) and 7 months to full recovery in women. This study also demonstrated a recurrence rate of 25% in men.^78^ Generally, reports suggest that based on the natural history of osteitis pubis, the average period—the condition may take to resolve—ranges from 3 to 12 months.^1^, ^26^, ^30^, ^77^

Only 5–10% of the cases are calcitrant to conventional therapies, and they usually develop chronic painful inflammatory course of the disease. Women can have a continuous vaginal discharge with chronic pelvic pain symptoms. These resistant cases may require further complex challenging surgical interventions that can end up with devastating complications.

## 9 CONCLUSION

Diagnosis of osteitis pubis is not straightforward because of its rarity. For all patients presenting with possible symptoms of osteitis pubis, a thorough history and meticulous physical examination should be performed. Laboratory tests are usually not of great help in most of cases. Osteitis pubis is typically a clinical diagnosis; however, MRI is currently considered as the most useful radiological imaging for confirming the diagnosis.

Treatment should ideally be planned in a multidisciplinary team inclusive of an orthopaedic surgeon with special interest in pelvic disorders or sports medicine specialist and physiotherapist particularly in difficult cases. Conservative management is usually effective in tackling the disease progression and improving the symptoms. Surgical interventions are needed only in severe cases and are not without long term complications. Complete recovery is achieved in most cases; however, some cases may end up with residual long‐term problems.

## CONFLICT OF INTEREST

None of the authors have any conflict of interest to declare.

## HUMAN AND ANIMAL RIGHTS

This article does not contain any studies with animals performed by any of the authors.

## Supporting information


**Figure S1**. Supporting Information
