# Supplementary material for: The challenges in diagnosis and management of osteitis pubis: An algorithm based on current evidence
Source: BJUI Compass. 2022 Mar 11;3(4):267–76. doi: 10.1002/bco2.127 (PMC9231671; doi:10.1002/bco2.127)
Supplement: Supplementary file 1 — Figure S1. Supporting Information [file BCO2-3-267-s001.docx]

**Algorithm of management of osteitis pubis:**

Second line of conservative management

Steroids+/-local anaesthetics

Contrast (symphyseography)
